# Neutrophil Extracellular Traps and Cancer: Trapping Our Attention with Their Involvement in Ovarian Cancer

**DOI:** 10.3390/ijms24065995

**Published:** 2023-03-22

**Authors:** María Castaño, Sarai Tomás-Pérez, Eva González-Cantó, Cristina Aghababyan, Andrea Mascarós-Martínez, Nuria Santonja, Alejandro Herreros-Pomares, Julia Oto, Pilar Medina, Martin Götte, Bárbara Andrea Mc Cormack, Josep Marí-Alexandre, Juan Gilabert-Estellés

**Affiliations:** 1 Haemostasis, Thrombosis, Arteriosclerosis and Vascular Biology Research Group, Medical Research Institute Hospital La Fe, 46026 Valencia, Spain; 2 Research Laboratory in Biomarkers in Reproduction, Gynaecology, and Obstetrics, Research Foundation of the General University Hospital of Valencia, 46014 Valencia, Spain; 3 Department of Obstetrics and Gynecology, General University Hospital of Valencia Consortium, 46014 Valencia, Spain; 4 Department of Pathology, General University Hospital of Valencia Consortium, 46014 Valencia, Spain; 5 Gynaecological Oncology Laboratory, Department of Oncology, KU Leuven, 3000 Leuven, Belgium; 6 Department of Gynecology and Obstetrics, Münster University Hospital, 48149 Münster, Germany; 7 Department of Pediatrics, Obstetrics, and Gynaecology, University of Valencia, 46014 Valencia, Spain

**Keywords:** neutrophils, neutrophil extracellular traps, NETs, cancer, ovarian cancer, NETosis, immunothrombosis, metastasis, Toll-like receptor 4, TLR4

## Abstract

Neutrophils, the most abundant circulating leukocytes, play a well-known role in defense against pathogens through phagocytosis and degranulation. However, a new mechanism involving the release of neutrophil extracellular traps (NETs) composed of DNA, histones, calprotectin, myeloperoxidase, and elastase, among others, has been described. The so-called NETosis process can occur through three different mechanisms: suicidal, vital, and mitochondrial NETosis. Apart from their role in immune defense, neutrophils and NETs have been involved in physiopathological conditions, highlighting immunothrombosis and cancer. Notably, neutrophils can either promote or inhibit tumor growth in the tumor microenvironment depending on cytokine signaling and epigenetic modifications. Several neutrophils’ pro-tumor strategies involving NETs have been documented, including pre-metastatic niche formation, increased survival, inhibition of the immune response, and resistance to oncologic therapies. In this review, we focus on ovarian cancer (OC), which remains the second most incidental but the most lethal gynecologic malignancy, partly due to the presence of metastasis, often omental, at diagnosis and the resistance to treatment. We deepen the state-of-the-art on the participation of NETs in OC metastasis establishment and progression and their involvement in resistance to chemo-, immuno-, and radiotherapies. Finally, we review the current literature on NETs in OC as diagnostic and/or prognostic markers, and their contribution to disease progression at early and advanced stages. The panoramic view provided in this article might pave the way for enhanced diagnostic and therapeutic strategies to improve the prognosis of cancer patients and, specifically, OC patients.

## 1. Introduction

Ovarian cancer (OC) remains the second most incidental but the most lethal gynecologic malignancy [1,2]. The American Cancer Society reported 21,410 new cases and 13,770 disease-related deaths in 2021, which represents 5% of female cancer deaths [1]. Epithelial ovarian carcinoma (EOC) is the most common form of OC [3] and is classified into four different histological subtypes: serous (including both high- and low-grade), mucinous, endometrioid, and clear cell OC [4]. The two other forms of OC are germ cell and sex cord-stromal, both comprising about 5% of all ovarian malignant tumors [5]. High-grade serous ovarian cancer OC (HGSOC) is the most common and aggressive form of EOC [6], with more than 80% of HGSOC patients diagnosed in advanced stages [7,8] where the 5-year overall survival drops from 92% in early-stages to 29% in advanced EOC [9].

When tumor extension and patient overall stage is compatible with primary debulking surgery, the gold-standard treatment for advanced EOC comprises initial cytoreductive surgery and postoperative first-line platinum- and taxane-based chemotherapy [10]. For patients in IIIC-IV stages or patients who are not suitable to undergo first-line cytoreductive surgery, either because of their clinical condition or because they have unresectable disease, neoadjuvant chemotherapy followed by interval cytoreductive surgery can be considered as an alternative approach [11]. However, most patients in advanced stages will develop chemoresistance and will eventually relapse, contributing to a poor prognosis. These data emphasize the need to deepen our understanding of the mechanisms of disease progression and the evaluation of alternative therapies.

As with many other cancers, metastasis is a challenge for patient survival. In OC, it begins once the cancer cells have detached from the primary ovarian tumor, individually or in clusters. Through a passive mechanism, cells are transported by the physiological movement of fluids to the metastatic niche [12]. Although systemic or lymphatic routes might explain the development of distant metastasis in the liver, lymph nodes, lung, bone, and brain, the ascitic fluid is involved in the common spread of OC cells through the peritoneal cavity [13]. Notably, HGSOC displays a metastatic tropism to the omentum [14]. Interestingly, several authors have shown that both inflammatory and immunosuppressive components of malignant ascites [12,15] and of the tumor immune microenvironment (TIME) [12,15,16,17] might contribute to this omental preference of detached OC cells.

Neutrophils, the most abundant circulating leukocytes, have been described as significant players of TIME primarily via neutrophil extracellular trap (NETs) formation. Thus, recent discoveries related to the mechanisms of cancer metastasis and progression have focused on this cell type, envisaging their potential pro-tumoral role [18,19,20,21].

Current OC research is primarily focused on identification of biomarkers to allow early detection of this pathology and on the development of innovative therapeutic approaches. However, few have ventured into the emerging hypothesis of a possible contribution of neutrophils and NETs to OC. Previous research highlights the importance of deepening this crosstalk and provides the background for the present work. In this review, we summarize the state of the art on the interplay between NETs and cancer, emphasizing OC. We aim to provide a panoramic view of the current knowledge on NETs and their involvement in cancer to finally focus on their relevance in OC, strengthening the perspectives opened by the data presented.

## 2. New Roles for Neutrophils

### 2.1. NETosis: A New Mechanism of Neutrophil Defense

To ensure the proper functioning of the organism, it is essential to count on a defense system capable of identifying pathogenic agents, differentiating them from self-components and eliminating them. This role relies on the immune system, constituted by a varied group of cell types that perform a coordinated response. The immune system is classified into innate and adaptive immunity. Effectors of innate immunity include macrophages, neutrophils, monocytes, eosinophils, basophils, and natural killer cells (NK). On the other hand, adaptive immunity involves T and B lymphocytes, which present a specific and structurally unique receptor [22]. While innate immunity is responsible for providing a rapid but non-specific response, adaptive immunity is specific and provides the organism with memory through the generation of antibodies that allow it to respond against a high diversity of antigens. Since the generation of a wide and varied repertoire of adaptive immune-specific molecules takes days, the first line of defense executed by innate immunity is crucial.

**Figure 1:**
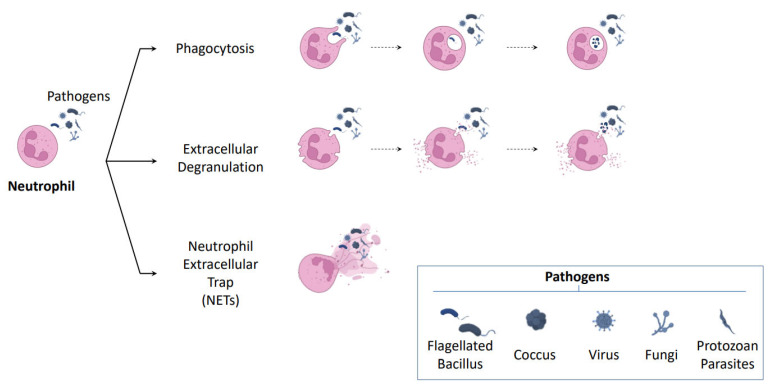
Pathogen elimination strategies conducted by neutrophils. The immune response is triggered by pathogens such as bacteria, fungi, viruses, and protozoan parasites. Available neutrophil strategies to achieve pathogen clearance include phagocytosis, extracellular degranulation, and neutrophil extracellular traps (NETs) release. Created with BioRender.com, accessed on 9 February 2023.

Neutrophils are the most abundant leukocytes and represent the first line of cellular defense, being, therefore, a key component of the innate immune response [23,24,25]. Although phagocytosis and degranulation have been traditionally seen as their main defense mechanisms, it has been widely described that activated neutrophils may also release NETs in a process called NETosis [26] (Figure 1). NETosis was identified for the first time in a seminal work by Brinkmann et al. in the early 2000s [27]; the study described NETs as three-dimensional structures composed of several granule and nuclear constituents, most notably DNA, neutrophil elastase (NE), and histones. Notably, these structures displayed antimicrobial activity by binding bacteria and killing them. Specifically, they demonstrated that NETs could act as a physical barrier that prevents further spread of pathogens. Moreover, they showed that these structures provide a high-local antimicrobial environment, highlighting the degradation of virulence factors by NE and the antimicrobial activity at low concentrations of nucleosomes. Since then, other authors have reported that NETs are also capable of trapping other pathogens such as fungi, viruses, and protozoan parasites [28,29,30]. At present, NETs are defined as web-like structures composed of DNA-histone complexes and cytoplasmic and granular proteins such as calprotectin, myeloperoxidase (MPO), and NE that, in addition to being involved in the defense against pathogens, may play a role in noninfectious pathologies such as obesity [31] and diabetes mellitus type II [32], atherosclerosis, thrombosis [33], psoriasis [34], systemic lupus erythematosus [35], rheumatoid arthritis [36,37], and cancer [38,39,40,41], among others. Although the NETosis process is by far the most studied, the release of extracellular traps (ETs) or ETosis has also been documented in other immune cells, including macrophages [42,43], mast cells [44,45], and eosinophils [46,47,48]. However, in contrast to the large number of studies focused on neutrophils, ETs in these cell types have been mainly identified in vitro models and their role has been mostly related to pathogen trapping and to a bactericidal capacity. Although evidence is much more limited, it is currently acknowledged that the inflammatory-inducing stimuli (lipopolysaccharides, inflammatory cytokines, damage-associated molecular patterns (DAMPs)), the ETosis types (suicidal, vital, and mitochondrial), and the composition of the traps (DNA, MPO, citrullinated histone 3 (citH3), proteases) do not markedly vary between ETs sources. Even so, NE, specific to the neutrophil cell type, is widely considered to be a key protein in NET formation and, consequently, could be used as one of the primary markers of these structures [49,50], potentially distinguishing the cellular origin of NETs from other ETs sources. Finally, it should be noted that neutrophils correspond to the most abundant leukocytes in the immune system, and it is likely that, in the presence of ETs, the greatest contribution comes from this cell type.

### 2.2. Mechanism of NETosis Formation

**Figure 2:**
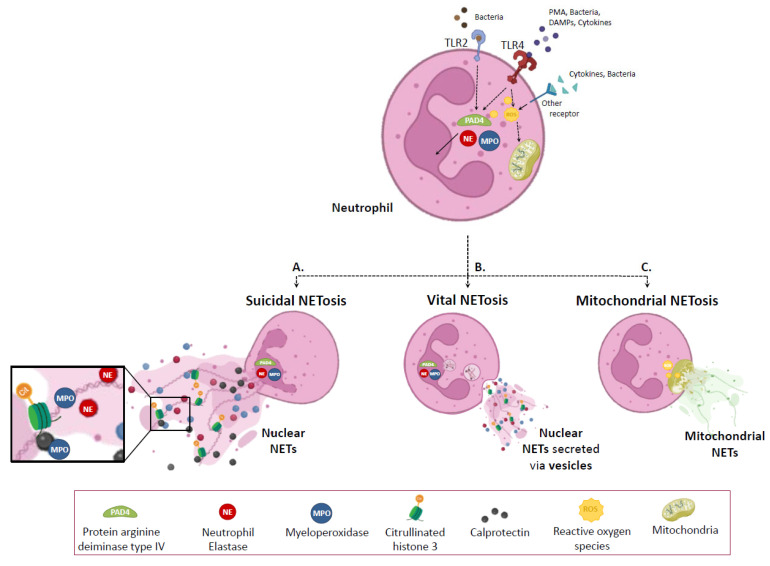
Types of NETosis. The neutrophil extracellular traps (NETs) formation and release may occur through three different processes: (**A**) suicidal, (**B**) vital, and (**C**) mitochondrial NETosis. Stimulus detection by the neutrophil membrane receptors triggers a signaling cascade. It activates Protein arginine deiminase type IV (PAD4), promotes the translocation of Neutrophil elastase (NE) and myeloperoxidase (MPO) to the nucleus, and could increases in Reactive oxygen species (ROS) levels. In nuclear NETs releases, PAD4 catalyzes histone 3 citrullination (citH3), while NE and MPO decondensed chromatin. PMA, phorbol myristate acetate; DAMPs, damage-associated molecular patterns; TLR2, Toll-like receptor 2; TLR4, Toll-like receptor 4. Created with BioRender.com accessed on 9 February 2023.

The steps and molecules involved in NETosis formation have been extensively studied. NETs formation may occur through at least three types of NETosis (Figure 2), giving rise to suicidal, vital, and mitochondrial NETosis. Interestingly, the evidence compiled to date suggests that the same cell type has the potential to undergo more than one mechanism of NETosis [51]. These different types of NETosis can be defined considering the origin of the extruded DNA, the inducer stimuli, the morphological changes undergone by neutrophils, and its viability after the process. 

On the one hand, suicidal NETosis was the first to be described. Currently, it has been stated that different stimuli and signaling pathways may be involved in this process. Independently of the activation pathway, this process is characterized by being detectable hours after cell activation and by ending with cell death because of the disruption of neutrophil’s plasma membrane during NETs extrusion. One of the best-described suicidal NETosis pathways involves the production of reactive oxygen species (ROS). Briefly, ROS can activate several key proteins involved in different parts of the process. ROS stimulates the release of MPO and NE from azurophilic granules into the cytosol, and thence to the nucleus to facilitate chromatin decondensation. ROS can also activate protein arginine deiminase type IV (PAD4), which mediates the conversion of arginine to citrulline on histones, inducing the loss of heterochromatin and multilobular nuclear structures [52]. Afterward, the nuclear envelope disrupts, and the chromatin is released into the cytosol, where it is decorated with other granular and cytosolic proteins such as calprotectin. This release of DNA into the cytosol implies remodeling of the lamin network by phosphorylation of lamin A via PKCα and cyclin-dependent kinase 4/6, after which mechanical forces resulting from chromatin expansion lead to the complete rupture of the nuclear envelope [53]. The whole process culminates with the release of NETs to the extracellular space and neutrophil death.

Deepening the knowledge of the NETosis process, recent research has shown that the Raf-MEK-ERK pathway is involved upstream of ROS production. In particular, it has been shown that the Raf-MEK-ERK pathway can modulate the NADPH oxidase and also affect the expression of the anti-apoptotic protein Mcl-1, which inhibits apoptosis and increases ROS to promote NETosis [54]. 

However, NETosis can also occur independently of NADPH [55] and ROS. A recently described ROS-independent pathway involves inflammasome activation as an additional mechanism regulating NETosis induction [56]. This inflammasome-dependent NETosis requires gasdermin D cleavage. Depending on the initial stimulus, cleavage and consequent activation of this pore-forming protein can be by NE or Caspase-11. In the second case, gasdermin D activation creates pores in the nuclear membrane that allow caspase-11 to access chromatin and mediate histones modifications [56,57], triggering an NE-independent NETosis.

In contrast, vital NETosis occurs minutes after cell activation. Its most remarkable feature is the maintenance of intracellular structures such as mitochondria and cell viability, and functions such as chemotaxis, adhesion, and phagocytosis during the process since NETs are released through vesicular transport and degranulation [58,59,60]. 

The third type was first described in 2009 [61] and was denominated mitochondrial NETosis. As for vital NETosis, this process was proven to be independent of cell death [59]. Consequently, NETs formed by living cells through mitochondrial NETosis contain mitochondrial DNA (mtDNA) released after neutrophil reactive species of oxygen (ROS)-dependent activation. 

As evidenced in the preceding paragraphs, the process of NETosis implies a certain complexity. Although the most studied pathway includes PAD4, NE, MPO, and histone 3 citrullination as hallmarks, it has been recently reported that it can be triggered by a variety of stimuli sensed by different receptors, can involve non-canonical pathways, and a number of types of histone post-translationals modifications such as acetylation [62].

### 2.3. NETosis and Thrombosis

One of the fields in which NETosis is gaining increasing focus is on the tight link between coagulation and the immune system, which has led to the coining of the term immunothrombosis. This concept has revealed the joint action of the coagulation proteins and the immune cells in venous thrombus formation [63]. Neutrophils play a central role in clot formation and degradation, primarily through NETs extrusion. A murine model of deep vein thrombosis (DVT) showed the appearance of a large clot in which neutrophils constitute the predominant leukocyte subset. Furthermore, neutrophil depletion resulted in a profound inhibition of DVT development [64]. Accordingly, patients with thrombosis have increased NETs markers in plasma, probably mediated by a decrease in the natural anticoagulant-activated protein C, which is known to inhibit NETosis [65]. Furthermore, neutrophils play an essential role in thrombus resolution, especially in the early stages [66]. All in all, NETs induce a pro-thrombotic state that has been correlated with many conditions and pathologies [67,68] and, vice versa, coagulation proteins can induce NETosis [69].

In the context of the SARS-COV-2 pandemic, research on thrombotic complications concerning COVID-19 has intensified. In two independent studies, patients hospitalized for COVID-19 were followed up for a minimum of 7 days and the incidence of thrombotic events was registered. In one of the studies [70], 49% of the 184 patients included in the trial presented thrombotic events, of which 87% were of the pulmonary embolism (Pe) type. In the other trial, that included 230 patients [71], the frequency of venous thromboembolism (VTE) was 26.5%, of which 74% were DVTs and 26% Pes. Overall, 20–50% of hospitalized patients with COVID-19 develop thrombotic complications. Based on the knowledge that viruses can trigger NETosis, the link between SARS-CoV-2 infection and NETs has been studied. Middleton et al. measured plasma MPO-DNA complexes and assessed NET formation ex vivo in COVID-19 neutrophils and healthy neutrophils incubated with COVID-19 plasma. Plasma MPO-DNA complexes increased in COVID-19 patients and illness severity correlated directly with plasma MPO-DNA complexes. COVID-19 neutrophils ex vivo displayed excessive NETs at baseline, and COVID-19 plasma triggered NET formation [72]. Li, Shaohua et al. [73] have documented an increase in the number of neutrophils in the circulation and lungs of infected patients, accompanied by increased levels of neutrophil-associated cytokines such as IL-8 and IL-6. Moreover, these neutrophils suffer an exaggerated NETosis when compared to those from uninfected patients; this allows them to correlate the number of neutrophils and their activation with disease severity. Accordingly, several studies have proved that the generation of NETs was higher in neutrophils from patients with COVID-19, leading to associated complications such as unfavorable coagulopathies and dysregulated immunothrombosis [72,74,75]. NETs formation was observed in both circulating and infiltrating neutrophils, causing lung lesions, extensive inflammation, thrombus formation, and, most interestingly, chronic aberrant immunity. This evidence supports the relationship between NETs, immunothrombosis, and COVID-19, along with its related disorders.

Thrombosis and cancer are two tightly related conditions. Thrombosis is the second leading cause of death in cancer due to patients’ high hypercoagulability and the occurrence of VTE, which is strongly related to lower survival [76]. Cancer cells promote a hypercoagulability state through multiple mechanisms, including the production of procoagulant and proaggregant molecules (e.g., tissue factor) and the release of pro-inflammatory cytokines that activate endothelial cells, platelets, and leukocytes [77]. This hypercoagulability state induces an increase in peripheral blood neutrophils prone to NETosis, and activates neutrophils to produce more NETs than those activated by other means [78]. Furthermore, NETs promote endothelial cell activation and increased thrombogenicity [79], all contributing to cancer-associated thrombosis. The primary tumor location is considered a risk of thrombosis in a wide variety of studies. Although the incidence of thrombotic events may vary between the different populations studied, Khorana and Gregory [80] have compiled the available information and reported that the tumor types with a higher frequency of thrombosis are pancreas (5.3–26%), stomach (6.8–13.6%), ovarian (5.2–25%), lung (1.8–13.6%), and brain tumors (1.6–26%).

NETosis has been found to be dysregulated in cancer-associated thrombosis. Thus, neutrophil activation markers in biofluids have been proposed as predictive thrombosis biomarkers to reinforce or substitute currently limited scores. For instance, citH3 has been proposed as a predictor for VTE events in cancer patients [81]. In pancreatic cancer patients, calprotectin measured at diagnosis has been proposed as a biomarker to predict future VTE events during follow-up (AUC = 0.77; 95% CI (0.57, 0.95)) [82]. In glioma, pre-surgical levels of cell-free DNA (cfDNA) and MPO have been proposed as predictors of incidental post-surgical pulmonary embolism (AUC = 0.71; 95% CI (0.52, 0.90)) [83]. All in all, the estimation of the thrombotic risk in cancer patients may allow a tailored thromboprophylaxis in dose and/or duration that may further avoid bleeding complications in low-risk patients.

## 3. Neutrophils in Cancer

### 3.1. Tumor Associated Neutrophils (TANs)

Current evidence suggests that neutrophils are actively attracted by chemokines to the tumor microenvironment (TME) [84,85,86] Chemokines are a crucial component of the TME as they enable cell-to-cell communication. Evidence indicates that, in established neoplasia, there is an increase in growth factors such as granulocyte and granulocyte-macrophage-colony-stimulating factor (G-CSF and GM-CSF, respectively) and inflammatory cytokines (like IL-6, IL-1β, and IL-17) produced not only by tumor cells but also by tumor-infiltrating leukocytes, macrophages, and neutrophils [87]. Cancer G-CSF [88] and endothelial IL-8 [89] are pointed out as the principal triggers of NETosis in tumors. Nevertheless, the full set of underlying factors responsible for TANs recruitment is diverse, and so is their role once they get there. 

In the TME, neutrophils can either promote or inhibit tumor growth depending on cytokine signaling, epigenetic modifications and other factors present in the TME that can modify the function and morphology of these cells.

### 3.2. Pro-Tumor Role of Neutrophils in Cancer

Deepening research established that neutrophils are an integral part of the TME and that the tumor can adapt the process they undergo to its advantage. Thus, neutrophils are potentially involved in tumor development, growth, and progression, undergoing a functional reassignment and adopting an immunosuppressive and pro-tumor status. 

The pro-tumorigenic action of neutrophils can be exerted in several ways. It has been demonstrated that this cell type up-regulates the levels of metalloproteinases and integrins, leading to extracellular matrix remodeling, favoring processes of tumor dissemination and vascularization of the metastatic focus. They can also interfere with the immune response either by preventing NK cell recognition upon transferring their major histocompatibility complex-I to tumor cells [90], recruiting antiinflammatory macrophages and T-regulatory cells, or suppressing CD8 T-cell function [91].

Within the TME, the interaction between tumor cells and TANs is crucial for tumor survival. As reviewed by Yu et al. [90], this interaction may include platelet activation by tumor cells. Specifically, platelet activation leads to the secretion of metastasis-trigger molecules, the presentation of immunoregulatory molecules on their surface, and promotes the adhesion of tumor cells and TANs. In this regard, membrane receptors have been shown to play a crucial role for both neutrophils and tumor cells, as they can sense TME molecules. Different specific receptors capable of recognizing DAMPs and pathogen-associated molecular patterns (PAMPs) are known, among which Toll-like receptors (TLRs) can be distinguished. 

#### Role of TLRs in Cancer

Of the 11 members of the TLRs family, Toll-like receptor 4 (TLR4) was the first to be discovered in humans. It is located in the cell membrane along with other TLRs, such as TLR5, TLR10, and heterodimers of TLR2 with TLR1 and TLR6 [92]. 

In neutrophils, TLR4 was shown to induce the expression of genes involved in inflammatory responses [93]. The activation of this receptor triggers a series of processes, including the production of ROS, TLR4-dependent NF-kβ, and PAD4 activation and degradation of the nuclear envelope leading to the release of DNA [94]; all these are processes involved in NETosis.

Although TLRs are primarily expressed in innate immune cells and participate in immune response regulation, it has been shown that they are also expressed in tumor cells. In particular, TLR4 is overexpressed in different metastatic tumor cells positively correlating with tumor cell survival, metastasis, and drug resistance [95]. In these cells, TLR4 expression has been primarily implicated as a mechanism to manipulate the TIME and achieve increased cell proliferation and tumor expansion. Several authors reported that TLR4 activation promoted the production of immunosuppressive and proangiogenic cytokines by tumor cells, including IL-10, IL-8, TGF-β, and vascular endothelial growth factor [91,96,97]. Moreover, TLRs expression and activation would also promote the epithelial-to-mesenchymal transition in tumor cells by upregulating metalloproteinases and activating NF-kβ pathway leading to tumor cell survival, proliferation, and migration [90]. Additionally, TLR4 has also been involved in chemotherapy-driven metastasis. Specifically, molecules released by dead cells due to chemotherapy activate TLR4, which induces tumor inflammation and upregulates survival proteins required for cell growth and tumor invasion [95,98,99]. 

Due to the relevant role of TLRs in the innate immune response and cancer [100], several strategies modulating TLRs have been explored [101]. On the one hand, the presence of these membrane receptors on immune cells renders them attractive targets to promote the induction of antitumor responses through agonists. To date, two agonist therapies have been evaluated. First, a phase I clinical trial using the Bacillus Calmette-Guérin (a strong immune adjuvant for cancer immunotherapy and a mixed TLR2/TLR4 agonist) [102] on 18 patients with different cancers refractory to standard therapies (7 melanoma, 5 colorectal, 4 hepatobiliary, 1 ovarian, and 1 lung cancer) showed that the compound was well tolerated and induced an appropriate immune response. In addition, the monophosphoryl lipid A, a TLR4 agonist, was approved by the Food and Drug Administration [103] and showed that metastatic macrophages can be reprogrammed to kill cancer cells in a murine model of luminal B breast cancer when administered intratumorally or intraperitoneally jointly with IFNγ. Motivated by these results, authors also evaluated its effect in an OC mouse model, observing that monophosphoryl lipid A plus IFNγ suppressed the metastatic progression of ovarian cancer, increased the median survival of the mice, and the percentage of monocytes in the ascites.

Nevertheless, it should be kept in mind that traditional antitumor therapies provoke an immunosuppressive state in patients, which challenges the immune enhancement effect of TLRs agonist strategies. Moreover, the risk of autoimmune diseases mediated by agonist administration should not be overlooked.

As previously mentioned, the overexpression of TLR4 on tumoral cells has been related to acquired chemoresistance, metastasis, and tumor cell survival. Hence, Kashani et al. [104] evaluated the effect of TAK-242, a TLR4 antagonist also known as resatorvid, in an OC cell line model. Authors found that co-treatment of paclitaxel and TAK-242 not only led to tumoral cell cycle arrest and apoptosis, but also satisfactorily decreased the expression of TLR4 and different interleukins in these cells. Nevertheless, the use of TLRs antagonists could also involve a greater susceptibility to opportunistic infections. 

All in all, the development of clinical cancer therapies, including immune adjuvants, still has a long way to go. Even though therapeutic options based on TLRs other than TLR4 have reached the stage of clinical trials for OC [105], there are still not enough to draw firm conclusions, since the expression of TLRs on immune cells and cancer cells seem to exert opposite effects. Further clinical trials are needed to elicit the overall effect of these drugs on humans.

### 3.3. Pro-Tumor strategies Involving NETs

**Figure 3:**
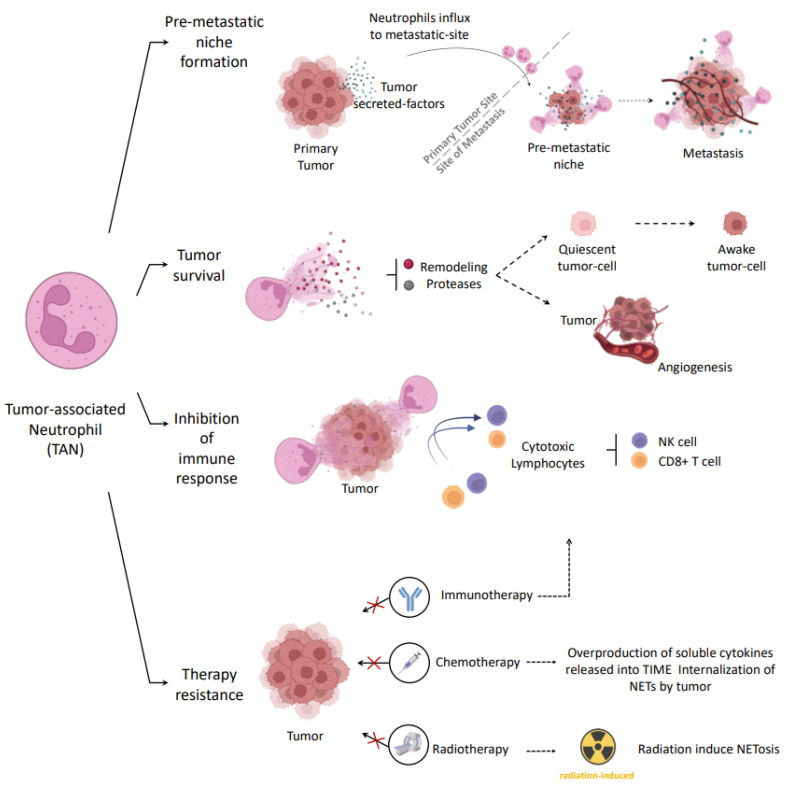
Pro-tumor role of NETs in cancer. Cancer cells recruit neutrophils to the tumor microenvironment and tumor-associated neutrophils (TANs) pro-tumor strategies could involve neutrophil extracellular traps (NETs). Those include pre-metastatic niche formation, promotion of tumor-survival processes, inhibition of the immune response, and therapy resistance. Created with BioRender.com accessed on 9 February 2023.

NETs formation has been widely reported in the TME of several cancers [85,106,107]. Tumors proved to be very efficient at taking advantage of these structures, which were initially expected to be responsible for their elimination. Neutrophils’ pro-tumor strategies involving NETs include: (1) pre-metastatic niche formation, (2) promotion of processes that favor tumor survival, (3) inhibition of the immune response, and (4) resistance to oncologic therapies (Figure 3).

Cancer progression and metastasis involves distant tissue colonization. It has been established that these focuses usually present a favorable microenvironment for the implantation of tumor cells before their arrival. This theory was postulated many years ago by Steven Paget and is known as “seed and soil” [108]. 

Based on this theory, recent evidence points out that primary tumors can release several factors to recruit neutrophils and induce NET release in pre-metastatic sites. Subsequently, formed NETs serve as scaffolds for circulating tumor cells and provide a favorable microenvironment for tumor growth and metastasis. Specifically, this mechanism has been proposed to explain the colonization of different types of cancer to target organs, such as that of OC to the omentum [109] and of colorectal, lung, and breast cancer to the liver [110,111,112]. 

Emerging evidence suggests that different NETs components may promote tumor progression, either by direct interaction with tumor cell receptors or indirectly by remodeling the intracellular matrix. For instance, NETs DNA-histones complexes can interact with CCDCD25 transmembrane protein in breast cancer cells leading to the activation of the ILK-pavin pathway to enhance cell motility [112]. For its part, NE may trigger TLR-4 signaling pathways in colorectal cancer cells, resulting in the upregulation of proteins involved in tumor mitochondrial biogenesis and growth [113]. On the other hand, it has been shown that NE and MMP9 proteases can awaken quiescent breast tumor cells through extracellular matrix remodeling. Specifically, proteases cleaved laminin, which activates α-3-β-1 integrin and consequently re-initiated cancer cell proliferation [114]. Interestingly, results suggest that these same proteases could be involved in tumoral angiogenesis by degrading cadherin from endothelial cells, thus promoting vascular permeability [115]. Given the importance of angiogenesis in tumor survival, studies linking NETs and angiogenesis in cancer are needed. 

Recent discoveries indicate that NETs may also act as a protective shield for tumor cells against cytotoxic immune cells. Specifically, Teijeira et al. [116] demonstrate in their extensive work that colon tumor spheroids and breast tumor cells in living mice can be coated with NETs. Moreover, these authors describe that NET structures may act as physical barriers that impair the arrival and contact of immune-cytotoxic cells (such as NK and CD8^+^ T-cells), protecting tumor cells from direct cytotoxicity and hampering their elimination. 

TIME plays a key role in resistance to cancer therapies. Components of this microenvironment, as TANs and consequently NETs, are involved in this resistance. Current approaches against cancer include chemo-, immuno-, and radiotherapies. Recent studies in multiple myeloma demonstrated a TAN-dependent chemoprotection driven by soluble factors released into the TME [117]. Extending these investigations, authors showed that NETs could be internalized by neoplastic cells and detoxify drugs such as doxorubicin [118]. Moreover, they showed that the treatment with DNase restored chemosensitivity in their animal models. Regarding immunotherapy, some studies have identified that the combined treatment of DNase or PAD inhibitors together with immune-checkpoints inhibitors would improve the results achieved by immunotherapy administration alone, suggesting that NETs may be interfering in the contact between cytotoxic and tumor cells [116,119]. Concerning radiotherapy, it has been suggested that the conventional radiotherapy itself would induce NETosis [120,121], although it is yet to be defined whether hadrontherapy has the capacity to induce this process. 

### 3.4. Anti-Tumor Role of Neutrophils in Cancer

As immune system effectors, leukocytes are presumably guided to infiltrate the tumor and to remain active in the surrounding regions as a defense. In solid tumors, neutrophils are part of the immune infiltrate and are known to communicate with macrophages and lymphoid cells, orchestrating their activation. In this sense, and in fulfilling their anti-tumor role, they guide and regulate the adaptive immune response [21]. Among the anti-tumor mechanisms are: (1) activation of adaptive immunity (T cell lymphocytes), (2) direct cytotoxicity (through the release of reactive nitrogen species and ROS, nitric oxide synthase expression, TNF-related apoptosis-inducing ligand, and TNF), and (3) triad interaction together with macrophages and T cells [87,122,123]. Based on the collected evidence, it seems that TANs usually participate in cellular networks that mediate anti-tumor activity in early carcinogenesis [21].

### 3.5. Anti-Tumor Strategies Involving NETs

Although current evidence primarily addresses the putative pro-tumorigenic role of NETs, occasional studies have identified that some anti-tumor functions of neutrophils may be mediated by the release of NETs. NETs can be involved in the adaptive immunity activation by priming T cells [124] or exert direct cytotoxicity on tumor cells through some of its components, such as MPO [125]. Moreover, in vitro assays in neck squamous carcinoma [126], melanoma [127], and colon cancer [128] cells suggest that NETs can interact with tumor cells and inhibit their migration and growth. 

Even in the face of this framework, the role of NETs in cancer progression remains controversial due to the different subtypes of neutrophils and their dual role. Therefore, the precise contribution of NETosis to cancer progression might be studied more deeply based on tumor type, stage, the inducing stimuli, cytokine profile, and tumor microenvironment.

## 4. NETs in Ovarian Cancer

OC, and especially HGSOC, is a complex disease for which multiple challenges remain. One of its main limitations is that it is primarily diagnosed in advanced stages, when most patients present widespread metastases throughout the peritoneal cavity. In this regard, although it may metastasize via systemic or lymphatic routes, most OC tumors spread following the peritoneal fluid dynamics. This characteristic way of metastasis turns peritoneal fluid into the most representative biofluid of the OC tumor environment and highlights the importance of broadening the knowledge about its components and processes. Furthermore, it suggests that the poor diagnostic performance of current markers and the lack of treatment response may be in part caused by the systemic approach on which both are based. 

Although NETs have received considerable attention in cancer research, studies in OC are still in their infancy. This review includes the current articles that consider the link between NETs to OC (Table 1).

Regarding diagnostic and/or prognostic markers, three studies have attempted to study the potential role of NETs markers in OC diagnosis or prognosis, though from different perspectives. On the one hand, Singel et al. [129] analyzed the levels of mtDNA, a mitochondrial damage-associated molecular pattern released by tumor cells during necrosis, and NE, as a marker of NETs granular content in ascites samples from patients with advanced EOC. Interestingly, mtDNA was considered a stimulus for NETosis activation. Survival analyses showed that mtDNA and NE levels positively correlated with reduced progression-free survival when the period was restricted to a 12-month window after surgery. Moreover, they demonstrated in vitro that ascites may attract neutrophils and induce NETosis, suggesting that mtDNA and other components present in this biofluid may activate neutrophil responses facilitating metastasis. Therefore, they proposed that these pathways would serve as potential prognostic markers and/or therapeutic targets. Using a similar approach, Muqaku et al. [130] generated multi-omics and fluorescence-activated cell sorting data from ascites samples of HGSOC patients. In their hands, ascites samples from patients with non-miliary metastases had increased levels of NET-associated molecules (NE and MPO) and local inflammatory markers (calprotectin heterodimer comprising S100A8 and S100A9, also considered as a cytoplasmic marker of NETs) when compared to ascites samples with miliary metastases. In contrast, these samples showed increased levels of systemic inflammation markers (such as C-reactive protein (CRP)). Contrary to what was previously described by Singel et al. [129], in this study, an increased ratio S100A8/CRP abundance was associated with favorable survival of HGSOC patients. Finally, Dobilas et al. [131] studied the discriminative potential of two NETs markers (double stranded DNA (ds-DNA) and citH3) in plasma samples from patients with ovarian tumors and compared it with the diagnostic ability of CA125, the most widely used clinical biomarker, to predict OC. In their study, only CA125 levels were increased in borderline and ovarian tumors when compared to benign tumors. Moreover, CA125 levels were associated with worse overall survival.

As previously stated, OC displays a metastatic tropism for the omentum. However, the molecular mechanisms that allow the targeted colonization of HGSOC to this tissue have not been elucidated. In a recent work conducted by Lee et al. [109], researchers suggested that early-stage ovarian tumors can release inflammatory factors to recruit neutrophils into the omentum and induce NETs secretion. Subsequently, disseminated cells through the PF would bind to the formed NETs to conform pre-metastatic implants and promote tumor metastasis. Remarkably, they observed NETs in the omentum of ovarian tumor-bearing mice and women with non-metastatic early-stage OC. Moreover, they described how genetic and pharmacological blockade of PAD4 expression and treatment with DNase notably decreased omental metastasis. Taken together, these results postulate that neutrophil influx into the omentum could be a prerequisite step to the establishment of OC pre-metastatic niches and suggest that the interruption of NETs formation could prevent omental metastasis.

Based on these results, our research group aimed to evaluate whether NETosis could also contribute to the advanced stages of OC, which correspond to more than 80% of cases. Thus, in a recent work [132], we quantified five biomarkers of NETosis (cfDNA, nucleosomes, citH3, calprotectin, and MPO) in plasma (systemic level) and PF (tumor environment) samples from women with advanced HGSOC and control women. Our results showed that an increased NETosis occurs in biofluids from advanced HSGOC patients, primarily in the tumor environment, potentially contributing to the progression of HSGOC. Moreover, we compared the levels of NETosis biomarkers between patients with and without neoadjuvant treatment, observing that systemic neoadjuvant treatment has a major influence on NETosis markers at the systemic level, but its effect is rather limited in the tumor environment. Although far from the scope of this work, we are aware that both radiotherapy and/or immunotherapy could modulate NETosis markers, which deserves further devoted studies. Should these findings be confirmed, these observations might pave the way for the improvement of the therapeutic landscape in advanced HGSOC. 

The high relapse rate associated with OC, primarily due to a lack of complete response to disease treatment, highlights the need of both to identify new therapeutic targets and to characterize putative mechanisms of resistance to treatment. In this regard, NETs have been proposed as possible structures involved in resistance to treatment [120]. In the context of OC, Tamura et al. [133] demonstrated in vitro that NETs can capture drugs such as doxorubicin and paclitaxel and interfere with their pharmacokinetics. Moreover, they showed that doxorubicin-NETs interaction reduced the apoptotic effect of doxorubicin, which was reversed by DNaseI administration. Thus, these researchers hypothesize that NETs may capture anticancer drugs, especially those with affinity to bind DNA, such as platinum, first-line neoplastic drugs for OC for which there is often resistance. Furthermore, they also propose that interfering with the formation or destruction of NETs could be a beneficial strategy to enhance the effect of this type of drug. This concept agrees with the fact that, to date, clinical trials on immunotherapies have presented modest responses in patients with EOC [134,135,136,137,138,139,140,141]. Although the low mutation burden of the tumor and the redundancy of immune-checkpoints have been blamed for the ability of tumor cells to overcome the blockade, recent findings suggest that the coating of OC cells by NETs might be also involved in immune-checkpoint blockade resistance in OC.

## 5. Conclusions

NETosis, a new mechanism of action of neutrophils, involves the release of NETs composed primarily of DNA, histones, calprotectin, MPO, and NE. Although initially described in the defense against pathogens, current knowledge involves them in physiopathological conditions such as immunothrombosis and cancer. In cancer, neutrophils and NETs are involved in pre-metastatic niche formation, increased survival, inhibition of the immune response, and resistance to oncologic therapies. 

Throughout this review, we have gathered evidence about the relationship between NETs and cancer and how this might lead to worse disease development. Consequently, NETs emerge as valuable candidates for targeting in cancer. Unfortunately, there is a lack of clinical trials in progress in this respect. Moreover, since NETosis involves neutrophils, the most abundant cells of the immune system, several experiments are still required in different models to define the best strategy without affecting their beneficial granulating and phagocytic functions, nor affecting established therapies by interfering with their targets. 

**Figure 4:**
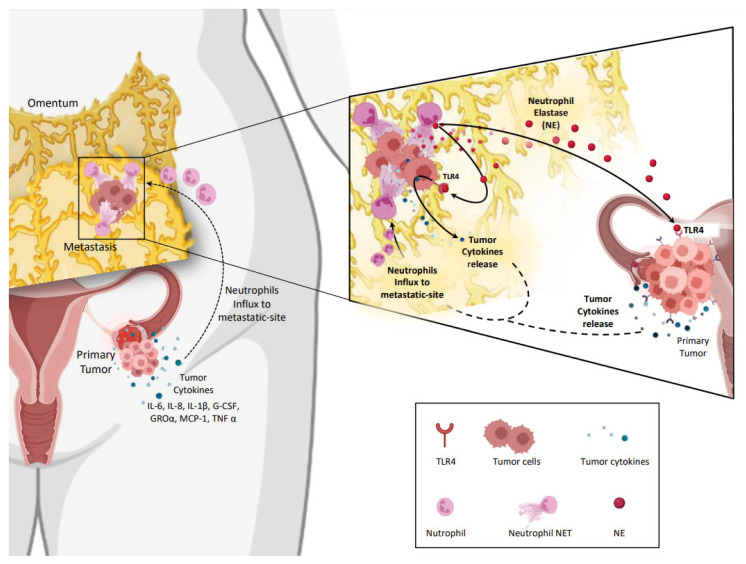
Putative positive feedback loop for ovarian cancer metastasis based on NETosis. Ovarian cancer (OC) cells release cytokines to attract neutrophils to pro-metastatic niches and to induce the release of neutrophil extracellular traps (NETs). In turn, NETosis releases neutrophil elastase (NE) to the tumor environment, acting on Toll-like receptor 4 (TLR4) which increases the release of tumor cytokines, forming a positive pro-metastatic feedback loop. Created with BioRender.com accessed on 9 February 2023.

Regarding OC, recent discoveries reveal a crucial pernicious role of NETs in this type of cancer, which remains the most lethal gynecologic malignancy and the second most incidental. Apart from their putative role as biomarkers, NETs have been involved in resistance to chemo-, immuno-, and radiotherapies and tumor progression in early and in advanced stages. From the evidence compiled in the literature regarding OC, a putative positive loop for OC metastasis based on NETosis can be established. We propose that, at the primary location, OC cells can release specific cytokines (i.e., IL-6, IL-8, IL-1β, G-CSF, GROα, MCP-1, and TNFα) to the tumor environment (i.e., peritoneal fluid) which may attract neutrophils to pro-metastatic niches (for instance, omentum) to induce NETosis. NETs on pro-metastatic niches can trap detached OC cells to initiate metastasis. In turn, released NE, among other factors, might spread through biofluids (for instance, peritoneal fluid) to reach TLR4 on tumor cells and activate intracellular signals that increase the release of pro-metastatic cytokines, forming a positive pro-metastatic feedback loop (Figure 4). In conclusion, a targeted therapy to disturb this positive loop might represent a novel therapeutic benefit for OC patients.

## Figures and Tables

**Table 1:** List of studies related to NETs in OC.

Authors [Refs.]	Year	Title	Experimental Design	Study Cohort/Sample	NETs Markers Measured	Type
Lee, et al. [109]	2019	Neutrophils facilitate ovarian cancer premetastatic niche formation in the omentum.	In vivo: Orthotopic tumors in immunocompetent C57BL/6 mice, analysis ovarian cancer cell implantation kinetics into omentum, neutrophil levels. In vitro: Stimulation of neutrophils with OC cells conditioned media, analysis of mice and human omental tissues.	*n* = 46 C57BL/6 mice *n* = 5 NSG mice *n* = 5 Nude mice *n* = 10 Omentum from patients without cancer *n* = 10 Omentum from patients with SLMP *n* = 10 Omentum from patients with HGSOC	DNA, citH3	Original Research
Singel, et al. [129]	2018	Mitochondrial DNA in the tumor microenvironment activates neutrophils and is associated with worse outcomes in patients with advanced epithelial ovarian cancer.	In vitro: NETs markers analysis in ascites samples from patients with advanced EOC, stimulation of healthy donor neutrophils and platelets.	*n* = 68 Ascites from patients with advanced EOC *n* = 5 Resected tumors from patients with advanced EOC	mtDNA, NE	Original Research
Muqaku, et al. [130]	2020	Neutrophil Extracellular Trap Formation Correlates with Favorable Overall Survival in High Grade Ovarian Cancer.	In vitro: Multi-omics and fluorescence-activated cell sorting data from ascites samples of HGSOC patients.	*n* = 18 Melanoma patients *n* = 25 HGSOC patients *n* = 36 HGSOC patients data from other papers	NE, MPO, calrpotectin	Original Research
Dobilas, et al. [131]	2022	Circulating markers of neutrophil extracellular traps (NETs) in patients with ovarian tumors.	In vitro: NETs markers analysis in plasma samples from patients with ovarian tumors.	*n* = 199 Patients admitted for primary surgery of adnexal masses	ds-DNA, citH3	Original Research
Tomás-Pérez, et al. [132]	2023	Increased levels of NETosis biomarkers in high-grade serous ovarian cancer patients’ biofluids: potential role in disease diagnosis and management.	In vitro: NETs markers analysis in plasma samples and ascites from women with advanced HGSOC and control women.	*n* = 45 Plasma and PF samples from HGSOC patients *n* = 40 Plasma and PF samples from control women	cfDNA, nucleosomes, citH3, calprotectin, MPO	Original Research
Tamura, et al. [133]	2022	Neutrophil extracellular traps (NETs) reduce the diffusion of doxorubicin which may attenuate its ability to induce apoptosis of ovarian cancer cells.	In vitro and ex vivo: Analysis of the effect of NETs on anti-cancer drugs pharmacokinetics.	*n* = N/A Blood samples from healthy patients *n* = N/A balb/c nude mice	N/A	Original Research

cfDNA: cell free DNA, citH3: citrullinated histone 3, ds-DNA: Double stranded DNA, EOC: Epithelial ovarian carcinoma, HGSOC: High grade serous ovarian cancer, MPO: myeloperoxidase, mtDNA: mitochondrial DNA, NE: Neutrophil elastase, NETs: Neutrophil extracellular traps, NSG mice: NOD scid gamma mice, N/A: not available, OC: Ovarian cancer, PF: peritoneal fluid, Refs.: reference number, SLMP: Serous low malignant potential, Type: type of article reviewed, Year: year of publication.

## Data Availability

Not applicable.
